# Analysis of Oncology and Radiation Therapy Representation on the National Board of Medical Examiners Official Practice Material for the United States National Standardized Medical Board Examinations

**DOI:** 10.1007/s13187-024-02475-0

**Published:** 2024-07-13

**Authors:** Mary T. Mahoney, Lauren C. Linkowski, Trudy C. Wu, Jie Jane Chen, Beth K. Neilsen, Petria S. Thompson, Michael D. Mix, Karna T. Sura, Malcolm D. Mattes

**Affiliations:** 1 Transitional Year Residency Program, Garnet Health Medical Center, 707 East Main St, Middletown, NY 10940 USA; 2 Department of Internal Medicine, University of Pennsylvania, Philadelphia, PA USA; 3 Department of Radiation Oncology, University of California Los Angeles, Los Angeles, CA USA; 4 Department of Radiation Oncology, University of California San Francisco, San Francisco, CA USA; 5 Department of Radiation Oncology, SUNY Upstate Medical University, Syracuse, NY USA; 6 Department of Radiation Oncology, Rutgers Cancer Institute of New Jersey, New Brunswick, NJ USA; 7 Department of Radiation Oncology, Fox Chase Cancer Center, Philadelphia, USA

**Keywords:** Radiation oncology, Undergraduate medical education, Standardized medical licensing examination, Oncology

## Abstract

Radiation therapy (RT) is a critical component of multidisciplinary cancer care, but has inconsistent curricular exposure. We characterize the radiation oncology (RO) content on the standardized undergraduate medical examinations by comparing its context and prevalence with other domains in oncology. National Board of Medical Examiners (NBME) self-assessments and sample questions for the United States Medical Licensing Exam (USMLE) Steps 1–3 and NBME clinical science shelf examinations were accessed (*n* = 3878). Questions were inductively analyzed for content pertaining to oncology and treatment modalities of RT, systemic therapy (ST), and surgical intervention (SI). Questions were coded using USMLE Physician Tasks/Competencies and thematic analysis. Descriptive statistics and analyses using the Kruskal–Wallis test are reported. A total of 337 questions (8.6%) within the USMLE and shelf exams included oncology content, with 101 questions (2.6%) referencing at least one cancer treatment modality (*n* = 35 RT, 45 ST, 57 SI). Treatment questions were more common on USMLE Step 2 CK (*n* = 35/101, 32%) compared to Step 1 (*n* = 23/101, 23%) and Step 3 (*n* = 8/101, 8%) (*p* < 0.001). RT was significantly less likely to be the correct answer (2/35, 6%) compared to ST (4/45, 9%) and SI (18/57, 32%) (*p* = 0.003). Therapeutic oncology questions are uncommon on the examination material, with an under-representation of radiation-related content, and contextual bias favoring surgical approaches. We advocate for greater RO involvement in the content creation of such examinations to help trainees better understand multidisciplinary cancer care.

**Supplementary Information:**

The online version contains supplementary material available at 10.1007/s13187-024-02475-0.

## Introduction

Radiation therapy (RT) is a critical component of multidisciplinary cancer care [1]. For patients with a wide variety of curable malignancies, advances in RT have led to significant improvements in patients’ quality of life through organ preservation by either enabling less extensive or morbid surgical procedures or obviating the need for surgery entirely [2–6]. For patients with metastatic disease, RT also improves quality of life by palliating symptomatic sites of disease or serving as a consolidative treatment to systemic therapy to prolong disease control [7–10].

In both the curative and palliative settings, a lack of knowledge among physicians about when to refer to radiation oncology (RO) may lead to delays or omission of RT, which can negatively impact patient outcomes [11–19]. This lack of knowledge may stem from both the siloed nature of graduate medical education (GME) in the US [20], as well as insufficient teaching during medical school on basic principles of clinical oncology and the effective inclusion of RO as one of the three pillars of multidisciplinary cancer care [21–24]. Ultimately, these deficiencies in knowledge can result in bias against RT, including possible missed referrals despite high-quality evidence, or incompletely informed patients when considering treatment options [11–19].

Standardized tests are an important driver of the content students learn during medical school [25]—as students spend substantial time preparing for these exams and most curricula are developed to ensure that, at a minimum, students learn enough to pass. The National Board of Medical Examiners (NBME) offers electronic self-assessments with practice questions retired from prior exams [26]. Students use these self-assessments to prepare for the United States Medical Licensing Exam (USMLE) Steps 1–3 series and NBME clinical science shelf examinations. There is evidence that the NBME self-assessments correlate positively with test performance, thus encouraging their widespread use among trainees [27–30]. Additionally, students and curriculum development committees alike, turn to the USMLE Content Outline [31] to inform necessary educational material for these exams.

Each of these resources contains content related to oncology. However, the distribution of questions related to carcinogenesis, pathophysiology, natural history, and oncologic treatment has not been described in the medical literature since a single qualitative study from the 1970s that predates the USMLE Step 1 exam [32]. To our knowledge, there has been no formal investigation on the oncology and therapeutic oncology topics that directly appear on US standardized medical exams. The purpose of this study was to better characterize the RO-related content on the standardized undergraduate medical examinations, and to compare its context and prevalence with that of the other domains in oncology, including medical and surgical oncology.

## Methods

### Materials

We electronically accessed all current and commercially accessible NBME published self-assessments for USMLE Step 1, Step 2 Clinical Knowledge (CK), Step 3, and relevant NBME Clinical Science subject examinations using the MyNBME℠ Examinee Portal [26]. Additionally, we electronically accessed all current and open-access USMLE and NBME Clinical Science sample questions [33, 34].

For the USMLE Step examination series, the self-assessments offered are the Comprehensive Basic Science Self-Assessment (CBSSA) for USMLE Step 1, Comprehensive Clinical Science Self-Assessment (CCSSA) for USMLE Step 2 CK, and Comprehensive Clinical Science Self-Assessment (CCMSA) for USMLE Step 3. The Clinical Subject Mastery Series (CSMS) for the NBME Clinical Science subject examinations, colloquially known as “shelf exams,” are composed of the Clinical Neurology Self-Assessment, Emergency Medicine Self-Assessment, Family Medicine Self-Assessment, Medicine Self-Assessment, Obstetrics and Gynecology Self-Assessment, Pediatrics Self-Assessment, Psychiatry Self-Assessment, and Surgery Self-Assessment.

For Step 1 practice questions, CBSSA Forms 25–30 (*n* = 1200) and USMLE Step 1 Sample Questions (*n* = 120) were included. For Step 2 practice questions, CCSSA Forms 9–12 (*n* = 800) and USMLE Step 2 CK Sample Questions (*n* = 120) were used. For Step 3 practice questions, CCMSA (*n* = 176) and USMLE Step 3 Sample Questions (*n* = 137) were available. In total, 2553 questions related to the USMLE Step Exams were included in this analysis.

For CSMS, only relevant shelf examinations that would reasonably cover oncological material were included, thus Psychiatry was omitted, leaving 1325 questions for analysis. Clinical Neurology Forms 3–6 (*n* = 200) were included along with practice questions (*n* = 20) for a total of 220 questions. Emergency Medicine Forms 1 and 2 were used (*n* = 100) along with the practice questions (*n* = 5) for a total of 105 questions. Family Medicine Forms 2 and 3 (*n* = 100) were included along with practice questions (*n* = 5) for a total of 105 questions. Internal Medicine Forms 5–8 (*n* = 200) were included along with practice questions (*n* = 20) for a total of 220 questions. Obstetrics and Gynecology Forms 3–6 were included along with practice questions (*n* = 20) for a total of 220 questions. Pediatrics Forms 5–8 (*n* = 200) were included along with practice questions (*n* = 20) for a total of 220 questions. Surgery Forms 5–8 (*n* = 200) were included along with practice questions (*n* = 20) for a total of 220 questions.

### Data Analysis

The questions were inductively analyzed for content pertaining to oncology and associated oncological treatment modalities. The criterion for coding the general oncology-related material was defined as any direct mention of, recognition of, or implied presence of a neoplastic condition in the question stem or correct response that was relevant to the published learning objective attached to the question. This included any questions that used neoplastic conditions as a basis of ethical or physician–patient communication prompts. This query formula is grossly in concordance with the precedent methodology of qualitative research on oncological material appearing on historical standardized undergraduate medical licensing examinations [32, 35]. We omitted questions where the neoplastic condition was not relevant to the learning objective (*n* = 279) or as the prompt of a biostatistics question (*n* = 24). Among the 2553 USMLE practice questions that were analyzed, there were 1320 USMLE Step 1 practice questions, 920 USMLE Step 2 CK practice questions, and 313 USMLE Step 3 practice questions. Additionally, 1325 practice questions were available for review from all included shelf exams.

We cataloged each item into three major categories: radiation therapy (RT), systemic therapy (ST), and surgical intervention (SI). RT was defined as ionizing radiation in the treatment of any neoplastic or appropriate benign condition for either curative or palliative intent. ST was an umbrella category of most pharmaceutical interventions with curative intent including chemotherapy, immunotherapy, or hormonal therapy used to treat a neoplastic condition. Of note, pharmaceuticals used in the palliative or hospice settings for pain management were not classified as ST and instead remained classified under oncology-related. SI was defined as any procedural intervention including excisional biopsy or resection as well as dermatological procedural interventions for cancer.

This classification was applied to every exam question in the dataset to find the relevant oncological and treatment modality questions, resulting in the creation of the oncology question pool. To characterize the oncology question pool, the content was analyzed according to the procedures of a grounded theory approach to qualitative research [36]. First, a single author read the full question stem, answer choices, and the provided explanations of questions within the oncology question pool. Without a prior framework to inductively analyze questions, the five core USMLE Physician Tasks/Competencies (medical knowledge, patient care diagnosis, patient care management, communication, professionalism) and thematic analysis principles were used to identify themes [37]. These themes became the framework illustrated in Supplemental Tables 1, 2, and 3. We then coded each question within the oncology question pool according to these themes. Once the codebook was finalized, two independent reviewers were assigned for each examination type to ensure the appropriateness of themes to the respective pool of oncological questions. The independent reviewers were residents in radiation oncology with prior experience in medical education pedagogy training and conducting qualitative research. The independent reviewers read the full question stem, answer choices, and the provided explanations, and then coded the questions according to the established codebook. If there was any disagreement between the two independent reviewers, a third independent reviewer would review the question and decide on the appropriate code.

Statistical Package for the Social Sciences (SPSS) Version 29 (IBM Corporation, Armonk, NY) was used for statistical analysis. Descriptive statistics are reported. Additionally, the agreement between each pair of independent reviewers for the USMLE Step 1, Step 2 CK, and Step 3 examination questions was measured using Cohen’s weighted Kappa statistics. The Kruskal–Wallis test was used in several subgroup analyses comparing oncology content on different exams, as well as the frequency of questions about each therapeutic modality. A *p*-value < 0.05 was considered statistically significant.

## Results

### General Oncology

There were a total of 337 unique oncology-related questions out of the 3878 questions assessed across all practice material (8.7%). Oncology questions were identified on every examination type evaluated. Among USMLE exams, there was a significantly higher frequency of oncology questions within the Step 1 questions (*n* = 132/1320, 10%) compared to Step 2 CK (*n* = 65/920, 7.1%) and Step 3 (*n* = 16/313, 5.1%) practice questions (*p* = 0.006). A total of 122 oncology questions were on the NBME shelf exams (9.2%). There was a higher frequency of oncology questions on the Surgical shelf material (*n* = 37/220, 17%), followed by Obstetrics and Gynecology (*n* = 25/220, 11%), Clinical Neurology (*n* = 22/220, 10%), Medicine (*n* = 20/220, 9.1%), and Pediatrics (*n* = 14/220, 6.3%). Oncology questions were uncommon on the Emergency Medicine shelf material (*n* = 2/105, 1.9%) and family medicine shelf material (*n* = 4/105, 3.8%).

The composition of questions organized according to the five core USMLE physician tasks/competencies is shown in Table 1. Patient care—diagnosis was the largest identified pattern on USMLE Step 1 (62/132, 47%) and Step 2 CK (39/65, 61%) oncology-related questions. Patient care—management was most common on USMLE Step 3 (9/16, 56%). Most of the shelf material also had patient care—diagnosis, which was the largest identified pattern on the Surgery (*n* = 23/37, 62%), Medicine (*n* = 13/20, 65%), Clinical Neurology (*n* = 16/22, 73%), and Pediatrics (*n* = 9/14, 64%) exams. Patient care—management was the largest identified pattern in Obstetrics and Gynecology (*n* = 13/25, 52%) and Family Medicine (*n* = 2/4, 50%).

**Table 1:** Frequency of oncology questions across all practice examinations

		Five core USMLE Physician Tasks/Competencies					Cohen Kappa (*p*-value^^^)
Practice exam name (total # questions)	Oncology questions *n* (%)	Medical knowledge *n* (%)	Patient care—diagnosis *n* (%)	Patient care—management *n* (%)	Communication *n* (%)	Practice based learning *n* (%)	
Step 1 (*n* = 1320)	132/1320(10%)	49 (37%)	62 (47%)	17 (13%)	7 (5.3%)	0 (0%)	0.73 (0.006)
Step 2 CK (*n* = 920)	65/920 (7.1%)	6 (9.4%)	39 (61%)	16 (25%)	3 (4.7%)	1 (1.6%)	0.474 (0.053)
Step 3 (*n* = 313)	16/313 (5.1%)	0 (0%)	7 (44%)	9 (56%)	0 (0%)	0 (0%)	0.722 (0.019)
Surgery (*n* = 220)	37/220 (17%)	2 (5.4%)	23 (62%)	9 (24%)	0 (0%)	3 (8.1%)	0.648 (0.014)
Obstetrics and gynecology (*n* = 220)	25/220 (11%)	1 (4.0%)	10 (40%)	13 (52%)	1 (4.0%)	0 (0%)	0.878 (< 0.001)
Clinical neurology (*n* = 220)	22/220 (10%)	4 (18%)	16 (73%)	2 (9.1%)	0 (0%)	0 (0%)	0.737 (0.012)
Medicine (*n* = 220)	20/220 (10%)	0 (0%)	13 (65%)	6 (30%)	0 (0%)	1 (5%)	0.615 (0.025)
Pediatrics (*n* = 220)	14/220 (6.4%)	1 (7.1%)	9 (64%)	2 (14%)	1 (7.1%)	1 (7.1%)	1.0 (0.001)
Family medicine (*n* = 105)	4/105 (3.8%)	0 (0%)	1 (25%)	2 (50%)	0 (0%)	1 (25%)	0.615 (0.025)
Emergency medicine (*n* = 105)	2/105 (1.9%)	0 (0%)	1 (50%)	0 (0%)	1 (50%)	0 (0%)	0.25 (0.54)

Distribution of oncology material across all practice examinations. Tabulation of oncology questions identified across all USMLE practice questions and NBME shelf practice questions, stratified within the five core USMLE Physician Tasks/Competencies [37], using the coding scheme provided in Supplemental Table 1

^^^The *p*-value corresponds to the inter-rater reliability, with a *p*-value < 0.05 set to be statistically significant

### Oncologic Therapeutic Modalities

**Fig. 1:**
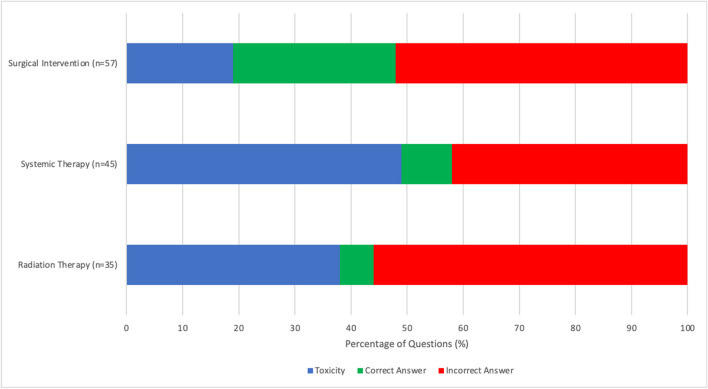
Comparison of therapies in oncology management related questions across all examinations by absolute question count. Comparison of the classifications of question types within the three therapies (radiation therapy, systemic therapy, and surgical interventions) for all USMLE practice questions and NBME shelf practice questions (*n* = 101) using the coding scheme of Supplemental Tables 2–3

Among the 3878 total questions and 337 oncology-related questions, 101 referenced at least one cancer therapy modality (2.6% and 30%, respectively). Sixty-six cancer therapy questions were on the USMLE Steps 1–3 examinations (65%), and 35 (35%) were on the NBME shelf exams. All three therapies—RO, ST, and SI—were represented on Surgery, Medicine, and Obstetrics and Gynecology practice shelf examinations. There were more therapeutic oncology questions within the practice questions for USMLE Step 2 CK (*n* = 35/101, 35%) compared to USMLE Step 1 (*n* = 23/101, 23%) and USMLE Step 3 (*n* = 8/101, 8%), which was statistically significant (*p* < 0.001). Questions involving SI had the highest frequency across all practice examination types. The classifications of question types within the three therapies are described and compared in Fig. 1.

RT questions tended to be focused on the toxicity of treatment (13/35, 37%), which was comparable to ST (22/45, 49%) but not SI (12/57, 21%) (*p* = 0.005). RT was more likely to appear as an incorrect answer choice (20/35, 57%) compared to ST (14/45, 31%) and SI (27/57, 47%) (*p* = 0.048). RT was significantly less likely to be the correct answer choice (2/35, 6%) when compared to ST (4/45, 9%) and SI (18/57, 32%) (*p* = 0.003). Although there were five mechanism of action questions for ST on USMLE Step 1, there were zero for RT or SI. Within the sampled clinical vignettes and explanations that discussed radiotherapy, we have identified some potentially inaccurate and misleading information, for which we also provided clarification and suggested accuracy improvements in Table 2.

**Table 2:** Findings of and proposed accuracy improvements for radiation therapy questions

Radiation therapy clinical vignette	Practice examination	Issue	Suggestion for improvement
RT toxicity of brachial plexopathy in post-mastectomy setting	Clinical Science Mastery Series (Neurology)	The NBME provided explanation states that brachial plexopathy in the post-mastectomy setting is a common side effect	The incidence of this toxicity is < 1%
RT as an answer choice to treating spinal cord compression in the setting of spinal cord metastasis	Clinical Science Mastery Series (Neurology)	The NBME explanation implies RT is a “definitive” therapy for this condition	Clarify that although RT may be indicated for urgent treatment, it is not a definitive treatment for metastatic spinal cord compression
RT as an incorrect answer (in favor of surgery) for the treatment of premalignant and malignant skin cancers, particularly of the face and eyelid	Clinical Science Mastery Series (medicine); Comprehensive Clinical Science (CCSSA)	RT may be the correct treatment rather than surgery. For example, surgery for eyelid tumors, especially large eyelid tumors, is deforming and it may be difficult to achieve negative margins	Provide a discussion of RT versus surgery
No indication for RT in the primary management of “rectosigmoid” cancer	Clinical Science Master Series (surgery)	There are different management guidelines depending on whether a malignancy is “rectal” or “sigmoid.” If sigmoid cancer, adjuvant RT would not be indicated while in rectal cancer, chemoradiation without surgery may be an option for patients who achieve a complete response following definitive chemoradiation	Replace “rectosigmoid” cancer in favor of delineation with “rectal” and “sigmoid” cancer. Explain why RT would be indicated or non-indicated for each type of cancer
No indication for RT in primary management of ovarian cancer	Comprehensive Clinical Science (CCSSA)	NBME explanation states that radiation use for ovarian cancer has little efficacy	Clarify that the reason that RT is not routinely used for ovarian cancer is that treatment to the abdominal cavity volume may result in toxicity, and not that there are efficacy concerns with RT for ovarian cancer
RT role in gastric mucosa-associated lymphoid tissue lymphoma of unknown mutational status	Comprehensive Clinical Science (CCSSA)	Whether antibiotics or RT is first-line therapy depends on the mutational status of the tumor	Provide the necessary clinical information in the clinical vignette to make an accurate selection of the correct therapy
RT induced bilateral pneumonitis from unilateral breast RT	Comprehensive Basic Science (CBSSA)	NBME explanation states that pneumonitis is a common side effect from RT	Provide the correct potential toxicity estimate based on anatomical considerations. First, pneumonitis is not a common side effect from RT. Second, if pneumonitis occurs, it would be unilateral after unilateral breast RT

Suggestions we propose to improve clinical vignettes and/or explanations that involved radiation therapy that was identified in the sampled NBME self-assessments for USMLE Step 1 (Comprehensive Basic Science), USMLE Step 2CK (Comprehensive Clinical Science), and NBME shelf examinations (Clinical Science Mastery Series)

## Discussion

Cancer is the second leading cause of mortality in the US, with a team of physicians spanning multiple specialties responsible for cancer prevention, screening, workup, management, and survivorship. As such, it is essential for medical school curricula and licensing exams to offer students a comprehensive and unbiased understanding of the basic principles of clinical oncology, so that trainees are prepared to serve their patients adequately. In this study, we have demonstrated how oncology is portrayed on national standardized testing materials by comprehensively evaluating commercially accessible NBME published practice material for USMLE Steps 1–3 and NBME shelf examinations. Among the three primary cancer treatment modalities, RT is more often portrayed negatively when compared to SI and ST. This introduces the potential for measurable bias in student learning, given that 37% of questions only mention RT in relation to toxicity, and 57% mention RT solely as the incorrect answer or second-line therapy relative to other treatment modalities. RT appeared as the correct answer in only 6% of questions, and this representation occurred exclusively on the USMLE Step 3 practice questions. Importantly, USMLE Step 3 occurs post-graduation, after most foundational medical learning has been completed. Based on these findings, we would advocate for greater involvement of radiation oncologists in the writing and/or review of the content of these examinations, as well as the questions included in the actual USMLE Step and NBME shelf examination questions, to provide a more accurate representation of RT and its role in multidisciplinary oncologic care.

Relatively few prior studies have addressed the composition of oncology content on undergraduate medical licensing exams. Similar to our findings, Baum et al. reported that oncology material represented 7–8% of NBME Part 1 questions [32]. Though there is certainly an extensive breadth of content to cover, the frequency of oncology-oriented questions seems low considering that cancer is nearly equal to heart disease as the leading cause of death in the US [38]. Interestingly, oncology-related content represents 30% of learning objectives on the Canadian equivalent exam, the Medical Council of Canada Qualifying Examination (MCCQE) Part I [35].

Furthermore, we believe that the imbalance between therapies tested on the exams, and the context in which each is discussed, may have significant potential to increase bias among students over time. This concern is particularly relevant in the context that there is substantial evidence that curricular exposure to clinical oncology is fragmented and often imbalanced at US medical schools [38, 39]. In totality, this supports the premise that a multidisciplinary effort to reform both oncology teaching and testing would be appropriate. Historically, there is precedent for leveraging USMLE content to drive curricular reform in medical schools. For example, governmental initiatives to enhance the nutrition education of US physicians in the 1980s and 1990s routinely suggested increasing the number of nutrition questions on National Board exams for medical students to thereby encourage medical schools to include nutrition in their curriculum [40–42]. Furthermore, the Undergraduate Medical Education Committee of the Society of Critical Care Medicine and the American Board of Obesity have used the presence of their respective topics on USMLE Content Outline and Licensing Examinations to advocate for a formalized curriculum within medical schools [43, 44].

Previous studies have demonstrated that a lack of topic expert involvement during multiple choice question (MCQ) item construction may negatively influence the balanced portrayal and accuracy of questions being asked on the USMLE and NBME examinations [40, 43]. RO representation is particularly low, as only one board-certified radiation oncologist is involved in content creation per the 2021 USMLE and NBME taskforce documents reports [45, 46]. A multidisciplinary expert approach for topic coverage, as was done for the construction of nutrition and obesity MCQ on NBME examinations, is indicated [40, 43].

The most important limitation of this work is that we were unable to assess the actual, currently tested content of the official USMLE Step and NBME examinations, and we relied upon the published practice exams as a surrogate. Future work could include repeating this study with the actual test questions from the USMLE Steps 1–3 and NBME shelf examination questions, to provide a baseline category of oncology and oncological therapies within these examinations. However, this is likely limited by the expiration time on the questions and their eligibility period. Another potential limitation is that the question reviewers for this study were not explicitly trained in multiple choice question (MCQ) construction or review, which is standard practice for all NBME test writers [47, 48]. However, all reviewers were resident physicians who have passed all USMLE and NBME shelf examinations. Our interpretations of the practice questions, particularly in regard to the five USMLE Physician Tasks and Competencies, may be different from the approach of trained NBME writers and reviewers. Their training is not publicized, nor available for review, limiting the similarity in mindset for approaching these questions.

## Conclusions

This study has demonstrated an inequitable and misleading portrayal of RT in comparison to other cancer treatment modalities on the NBME self-assessments and official practice questions for USMLE Steps 1–3 licensing and NBME shelf examinations. We advocate for greater RO involvement in the writing and/or review of the content of the USMLE Step and NBME shelf examination questions, to help trainees better understand the scope and role of RO in advancing patient care.

## Supplementary Information

Below is the link to the electronic supplementary material.

Supplementary file1 (DOCX 8 KB)

Supplementary file2 (DOCX 7 KB)

Supplementary file3 (DOCX 8 KB)

## Data Availability

This study was based on the questions derived from the commercially accessible NBME published self-assessments for USMLE Step 1, Step 2 Clinical Knowledge, Step 3, and relevant clinical science shelf examinations as well as open access USMLE and NBME Clinical Science sample questions. As such, the authors do not own these data and hence are not permitted to share them in the original form (only in aggregate form, e.g., publications). The aggregated, coded data that support the findings of this study are available from the statistical analysis author, MTM, upon reasonable request.
