# Supplementary material for: Analysis of Oncology and Radiation Therapy Representation on the National Board of Medical Examiners Official Practice Material for the United States National Standardized Medical Board Examinations
Source: J Cancer Educ. 2024 Jul 13;40(1):79–87. doi: 10.1007/s13187-024-02475-0 (PMC11846759; doi:10.1007/s13187-024-02475-0)
Supplement: Supplementary file 1 — Supplementary file1 (DOCX 8 KB) [file 13187_2024_2475_MOESM1_ESM.docx]

Supplemental Table 1. Codebook for General Oncology Questions for All Practice Examination Material

| USMLE Physician Tasks/Competencies | Selected Examples of Topics Relevant to Oncology |
| --- | --- |
| (1) Medical Knowledge/Scientific Concepts | Applying Foundational Science Concepts:   1. Pathophysiology: T-lymphocyte leukemia from gene therapy, drug resistance by drug efflux pump 2. Anatomy: Lymphatic drainage of a tumor, focal neurological sequelae of tumor location, fascial planes needed to be dissected for surgical removal 3. Genetics Syndromes: Neurocutaneous syndromes |
| (2) Patient Care - Diagnosis | History and Physical Examination:   1. Reported postmenopausal bleeding 2. Melanoma by photograph of gross pathology   Laboratory and diagnostic studies:   1. Lambert-Eaton Syndrome of small cell lung cancer. 2. Histological slide of renal cell carcinoma from a biopsy   Diagnosis: Making a diagnosis of small cell lung cancer from a constellation of evidence including a history of hemoptysis, radiographic depiction of a central lung lesion  Prognosis/Outcome: Metastatic disease |
| (3) Patient Care - Management | Prognostic Factors: Tumor grading and staging  Risk Factors: Smoking, cryptorchidism for testicular cancer, Schistosoma haematobium infection for bladder cancer  Preventative Interventions and Screenings: Awareness of the United States Preventive Service Task Force (USPSTF) guidelines for pap smears, colonoscopy, vaccinations, and mammograms.  Pain Management: Increasing narcotic dose to manage cancer pain  Clinical Intervention: Tissue biopsy |
| (4) Communication | Breaking bad news, discussion concerning physical examination findings, shared decision making, right to know in pediatric population |
| (5) Practice-Based Learning (PBL) | Understanding the applications of epidemiology |

**Supplemental Table 1** denotes the five core USMLE Physician Tasks/Competencies [37] and how recurring themes within oncology questions between all examination types fit into this rubric.
