# Supplementary material for: Analysis of Oncology and Radiation Therapy Representation on the National Board of Medical Examiners Official Practice Material for the United States National Standardized Medical Board Examinations
Source: J Cancer Educ. 2024 Jul 13;40(1):79–87. doi: 10.1007/s13187-024-02475-0 (PMC11846759; doi:10.1007/s13187-024-02475-0)
Supplement: Supplementary file 2 — Supplementary file2 (DOCX 7 KB) [file 13187_2024_2475_MOESM2_ESM.docx]

Supplemental Table 2. Coding Scheme for Therapeutic Modalities for USMLE Step 1 Examination Practice Material

| Pattern | Radiation Therapy (RT) | Systemic Therapy (ST) | Surgical Intervention (SI) |
| --- | --- | --- | --- |
| Mechanism of action of Therapy | 1 | 2 | 3 |
| Toxicity of Therapy | 4 | 5 | 6 |

**Supplemental Table 2.** Coding scheme used for USMLE Step 1 Examination Practice Material questions. The patterns of “Therapy of Mechanism of Action” and “Toxicity of Therapy” were identified from recurring themes of the oncology questions on the practice material.
