# Supplementary material for: Analysis of Oncology and Radiation Therapy Representation on the National Board of Medical Examiners Official Practice Material for the United States National Standardized Medical Board Examinations
Source: J Cancer Educ. 2024 Jul 13;40(1):79–87. doi: 10.1007/s13187-024-02475-0 (PMC11846759; doi:10.1007/s13187-024-02475-0)
Supplement: Supplementary file 3 — Supplementary file3 (DOCX 8 KB) [file 13187_2024_2475_MOESM3_ESM.docx]

Supplemental Table 3. Coding Scheme for Therapeutic Modalities for Clinical Science Practice Material

| Pattern | Radiation Therapy (RT) | Systemic Therapy (ST) | Surgical Intervention (SI) |
| --- | --- | --- | --- |
| Toxicity of Therapy | 1 | 2 | 3 |
| Therapy was Correct Answer to “Next Best Step” | 4 | 5 | 6 |
| Therapy was Incorrect Answer to “Next Best Step” Type 1* | 7 | 8 | 9 |
| Therapy was Incorrect Answer to “Next Best Step” Type 2** | 10 | 11 | 12 |

**Supplemental Table 3**. Coding Scheme used for USMLE Step 2CK, USMLE Step 3, and Clinical Science Mastery Series (‘shelf’) practice material questions. The patterns of “Toxicity of Therapy”, “Next Best Step (NBS) correct answer”, “NBS Wrong Answer Type 1”, and “NBS Wrong Answer Type 2” were identified using ground therapy.

*Denotes the particular therapy was the incorrect answer to a prompt of best next step as the therapy was (1) not indicted (2) not appropriate or (3) misleading answer

**Denotes the particular therapy was the incorrect answer to a prompt of best next step as this therapy was considered to be inferior or second line therapy to another therapy listed as an answer choice.
